# Role of Exercise Therapy in Prevention of Decline in Aging Muscle Function: Glucocorticoid Myopathy and Unloading

**DOI:** 10.1155/2012/172492

**Published:** 2012-06-17

**Authors:** Teet Seene, Priit Kaasik

**Affiliations:** Institute of Exercise Biology and Physiotherapy, University of Tartu, Ravila 14a, 50411 Tartu, Estonia

## Abstract

Changes in skeletal muscle quantity and quality lead to disability in the aging population. Physiological changes in aging skeletal muscle are associated with a decline in mass, strength, and inability to maintain balance. Glucocorticoids, which are in wide exploitation in various clinical scenarios, lead to the loss of the myofibrillar apparatus, changes in the extracellular matrix, and a decrease in muscle strength and motor activity, particularly in the elderly. Exercise therapy has shown to be a useful tool for the prevention of different diseases, including glucocorticoid myopathy and muscle unloading in the elderly. The purpose of the paper is to discuss the possibilities of using exercise therapy in the prevention of glucocorticoid caused myopathy and unloading in the elderly and to describe relationships between the muscle contractile apparatus and the extracellular matrix in different types of aging muscles.

## 1. Introduction

Aging is a multifactorial process influenced by biological, physiological, psychological, and social changes. The biological and physiological changes are primarily associated with a decline in muscle mass, strength, endurance and the inability to maintain balance [1–3]. Physical risk factors for falling, such as muscle weakness and an inability to maintain static or dynamic balance, lead to severe injury in the elderly [3]. Changes in skeletal muscle quantity and quality lead to disability in the aging population [4].

The rate of muscle loss has been estimated to range from 1% to 2% per year past the age of 50, as a result of which 25% of people under the age of 70 and 40% over the age of 80 are sarcopenic [5, 6]. In both young and aged skeletal muscle, it has been shown that oxidative stress increases in response to unloading [7] and may have an important role in mediating muscle atrophy [8]. It has also been proposed that changes caused by aging and unloading are muscle specific [9]. Muscle unloading results in a decrease in the number of myonuclei and an increase in the number of apoptotic myonuclei in skeletal muscle [10]. Heat-shock protein (HSP) 70 inhibits caspase-dependent and caspase-independent apoptotic pathways and may function in the regulation of muscle size by inhibition of necrotic muscle fiber distribution and apoptosis in aged muscle [11, 12]. The decline of muscle mass is primarily caused by type II fiber atrophy and loss in the number of muscle fibers. Increased variability in fiber size, accumulation of nongrouping, scattered and angulated fibers, and expansion of extracellular space are characteristic to muscle atrophy [13, 14]. Loss of fiber number as well as decreased production of anabolic hormones, for example, testosterone, growth hormone, insulin-like growth factor 1 (IGF-1), and an increase in the release of catabolic agents are principal causes of sarcopenia, and interleukin-6 has also been shown to amplify the rate of muscle wasting [15, 16].

Aging skeletal muscle becomes less powerful, fat is redistributed from the depot to muscle [17], and altered collagen synthesis and posttranslational changes in the structure of collagen reduce the elasticity of ligaments [18, 19].

The properties of muscle strength and stiffness that control balance between the ability of muscle fibers to resist stretching depend on the degree of cross-linking of collagen molecules. With age, the number of cross-links increases and makes the collagen fibers too stiff for optimal function [20].

Skeletal muscle reloading after unloading has been shown to increase the recovery of motor activity, which is as fast as the recovery of muscle strength, but mechanical properties depend on the metabolism and regeneration of the muscle structures from disuse atrophy [4]. The qualitative remodeling of contractile proteins plays a certain role in impaired locomotion and general weakness in aging. Thus, when atrophic muscle becomes active again, muscle mass increases in a relatively short period of time but the recovery of muscle strength takes much longer [21].

Dexamethasone treatment increased aging muscle wasting much more than in the young [22, 23] and the main reason is the loss of myofibrillar proteins from muscle [22, 24]. The catabolic action of glucocorticoids on skeletal muscle was found to depend on the functional activity of muscle [25, 26]. Exercise with simultaneous glucocorticoid treatment is an effective measure in retarding skeletal muscle atrophy [27, 28] and provides protection against one of the major effects of glucocorticoid-muscle wasting [29]. The search for possibilities to rehabilitate the loss of physical function by exercise therapy in the elderly to prevent diseases is one of the challenges nowadays caused by the increase in the number of aging people in the society. The capacity to evoke structural and functional rearrangements in aging skeletal muscle depends on the oxidative potential of the fibers [30]. The integral indicator of muscle protein metabolism, the turnover rate, provides a mechanism by which strength exercise can change the renewal of contractile proteins in accordance with the needs of muscle contractile apparatus [31]. As oxidative capacity of skeletal muscle decreases in the elderly, endurance exercises seems to be effective in its restoration as it stimulates mitochondrial biogenesis and improves their functional parameters [32, 33]. Both, strength and endurance exercise seem to be promising tools for aging-related disease prevention.

In the present paper, we will discuss recent evidence of exercise therapy in the prevention of glucocorticoid caused myopathy and in case of skeletal muscle unloading, what is characteristic in aging population. We will describe the relationships between the muscle contractile apparatus and the extracellular matrix (ECM) in different types of aging muscle and changes in muscle strength, endurance, and motor activity.

## 2. Glucocorticoid-Caused Myopathy in Aging Muscle

**Figure 1:**
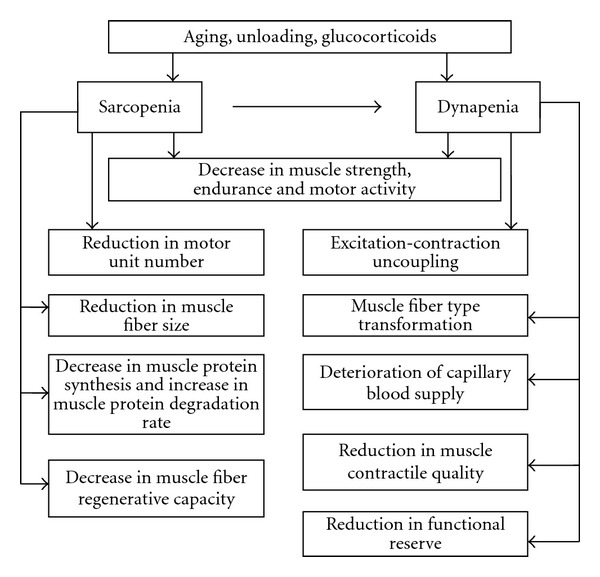
Effects of aging, unloading, and glucocorticoid treatment on skeletal muscle quantity and quality.

The anti-inflammatory effect of glucocorticoids is the reason for their wide use in various clinical scenarios. A side effect of glucocorticoids is muscle atrophy (Figure 1). It is well known that glucocorticoid-caused myopathy as well as Cushing's disease lead to a marked reduction in muscle mass, wasting of muscle, loss of strength, and selective atrophy of fast-twitch (FT) muscle fibers [34].

Aging-caused sarcopenia is associated with muscle weakness and impaired locomotion. Dexamethasone treatment significantly decreases muscle strength and motor activity of laboratory animals [23] and humans [22]. The reduced muscle mass in aging and dexamethasone treatment reflect a loss of myofibrillar proteins [4, 22, 24]. In both laboratory animals and humans, the synthesis rate of myofibrillar but not of sarcoplasmatic proteins decreases with age [22]. The treatment of adult and aged laboratory animals with dexamethasone leads to muscle wasting, but this effect was much more rapid in aged animals [35]. One of the reasons for this is that glucocorticoids decrease the stimulatory effect of insulin and IGF-1 in the skeletal muscle of old rats twice as much as in adults [36] and increase the expression of myostatin, a negative regulator of skeletal muscle [37]. In FT muscle fibers, an excess of glucocorticoids causes a break-down of thick and thin myofilaments and disintegration of individual myofibrils [38].

### 2.1. Role of Cellular and Extracellular Compartments in Changes of Muscle Strength and Motor Activity in Aging Myopathic Muscle

One of the important consequences of aging is impaired locomotion and general weakness [22]. Daily motor activity of old rats has a tendency to decrease in comparison with young rats. Dexamethasone treatment significantly reduced daily motor activity in both young- and old-age groups [39]. It has been shown there is a decline in muscle strength in atrophic muscle [40]. The qualitative remodeling of contractile proteins probably plays a certain role in this. Dexamethasone treatment decreased grip strength in both age groups but significantly more in the old than in the young group [23, 39]. It seems that in the everyday life of senescent rats, a decrease in muscle strength plays a more important role than daily motor activity.

The intensity of regeneration depends on the number of satellite cells under the basal lamina of muscle fibers, the size of the muscle, the type of injury, and the twitch characteristics of the muscle [30, 41–43]. Autografting of gastrocnemius muscle in old animals shows that regeneration proceeds significantly more slowly in comparison with young animals. Dexamethasone treatment decreased regeneration capacity both in young and old animals. Slower regeneration in old animals and after dexamethasone treatment in young and old groups is in good correlation with the decreased number of satellite cells [39]. Previous work has shown that dexamethasone treatment caused destructive changes in satellite cells on the ultrastructural level, which are similar to mother cell damage [38]. Myosin heavy chain (MyHC) composition during regeneration shifts from fast to slower type and this process is regulated by the cycle of denervation and reinnervation in regenerating skeletal muscle fibers [41].

Dexamethasone treatment led to quite similar results both in the young and the old but these changes are more significant in the aging group. Both aging and dexamethasone induced sarcopenic muscles have diminished regenerative capacity [39].

An excess of glucocorticoids has some similarities in its effect on the intracellular and extracellular compartments of skeletal muscle, for example, the decreased synthesis of proteins [34, 44]. The downregulation of collagen synthesis during dexamethasone administration shows that ECM components decrease [44]. As shown previously [38], MyHC synthesis rate only decreased in FT muscles, while the expression of collagen I, III, and IV mRNA decreased in both FT and slow-twitch (ST) muscles. It seems that dexamethasone treatment similarly influences fibril- and network-forming collagen expression in ST and FT muscles but differs at this point from contractile protein myosin synthesis, which was depressed only in FT muscles [45].

The second principal difference between contractile proteins and ECM during an excess of glucocorticoids is the degradation rate of proteins. Dexamethasone-increased the degradation of contractile proteins in skeletal muscle about two times but the expression of matrix metalloproteinase-2 (MMP-2) mRNA did not simultaneously significantly change although the degradation of collagens occurs mainly through MMP activity [46]. This is surprising as it was shown earlier that ST muscles contain significantly more collagen than FT muscles [47].

The concentration of endomysial collagen is higher around FT fibers [48], but as the downregulation of synthesis and unchanged degradation of type IV collagen are very similar to fibrillar collagen, and this shows that mechanical stability in skeletal muscle fibers, which is ensured by collagen IV, does not differ between ST and FT fibers. The effect of glucocorticoids on muscle weakness is applied through damaged contractile machinery of FT muscle fibers' intracellular compartment [23, 45].

### 2.2. Regeneration Capacity of Aging Myopathic Muscle

It has been shown that the turnover rate of contractile proteins in aging animals [49] and in young adults after the infusion of glucocorticoids decreases [23], and precursor cells required for muscle regrowth are morphologically and functionally damaged [38]. These changes together may be one of the reasons for sarcopenia (Figure 1). The mechanisms responsible for sarcopenia in aged skeletal muscle are largely unknown, but muscle satellite cells required for the repair of fibers certainly exhibit impaired activation [50] and proliferation [51] compared to young muscle.

Autografting of skeletal muscle has been used as a model of muscle regeneration. Higher oxidative capacity of muscle tends to ensure its faster regeneration [52–54]. It has been shown that the synthesis rate of contractile proteins depends on muscle oxidative potential [55]. In aging rats, the MyHC and actin synthesis rates decrease by about 30% and 23%, respectively [39]. It is known that aging is related to a dramatically reduced MyHC synthesis rate [56] without any change in MyHC on the transcriptional level [57]. Muscle wasting is also associated with increased protein degradation, particularly that of contractile proteins (Figure 1). Accumulation of abnormal proteins during aging is believed to result from defects in protein breakdown but very few experimental data support this hypothesis [22]. Results show that the degradation rate of contractile proteins in skeletal muscle during aging increased about two times, and dexamethasone treatment significantly increased the degradation rate in both age groups [39]. Previous works have shown that dexamethasone associated degradation starts from the periphery of myofibrils in muscle fibers with low oxidative potential [38]. This destruction process starts from myosin filaments and thereafter spreads all over the myofibrillar apparatus [38]. It has been shown that contractile proteins turned over slowly in old animals and subjects as well as in young rodents after dexamethasone treatment [34].

## 3. Effect of Unloading on Aging Skeletal Muscle

Aging is associated with a decline in skeletal muscle mass (sarcopenia), strength (dynapenia), and endurance (Figure 1). The term dynapenia was used to describe the age-related loss of muscle strength by Clark and Manini [58, 59]. Muscle unloading as a result of sedentary lifestyle, bed rest, spaceflight, and hindlimb suspension lead the skeletal muscle to microcirculatory disturbances, atrophy, protein loss, changes in contractile properties, and fiber-type switching [60].

The gradual development of functional limitations over an extended period of time is affected by the natural age-related decline in physical and biological properties, which already starts in midlife and increases the risk for a decline in physical functioning in later life [61].

During aging, the physical system suffers to a different extent and rate in diverse parts of the body. This results in reduced functional reserve, a decrease in vital capacity, deterioration of the capillary blood supply, and a decrease in muscle mass [62].

Living a sedentary life in older age, inactivity can lead to a loss of functional health due to deficits in muscle strength, endurance and flexibility [62]. “Use it or lose it” has proved to be a key rule for maintaining physical independence in the elderly [63]. One of the reasons for the development of muscle weakness in the elderly is decreased physical activity. Inactivity and aging cause a marked relative increase in the endo- and perimysial connective tissue, which results in changes in the mechanical properties of skeletal muscle [64]. Myofibrillar basal lamina becomes thicker and more rigid with age and increased cross-linking of collagen molecules make fibrils more resistant to degradation by collagenase [65]. The muscle tissue response to unloading seems to be more expressed than the connective tissue response [18, 66]. The connective structures are protected from rapid changes in tissue mass while muscle, which is known to act as a protein store for the organism, is subject to substantial and fast changes in tissue mass. Despite the small changes in connective tissue mass, important changes occur in the tissue structures during unloading and aging [4], which lead to the development of muscle weakness in case of restricted physical activity in the elderly.

## 4. The Preventive Role of Exercise Therapy on Aging Unloaded and Myopathic Muscle

**Figure 2:**
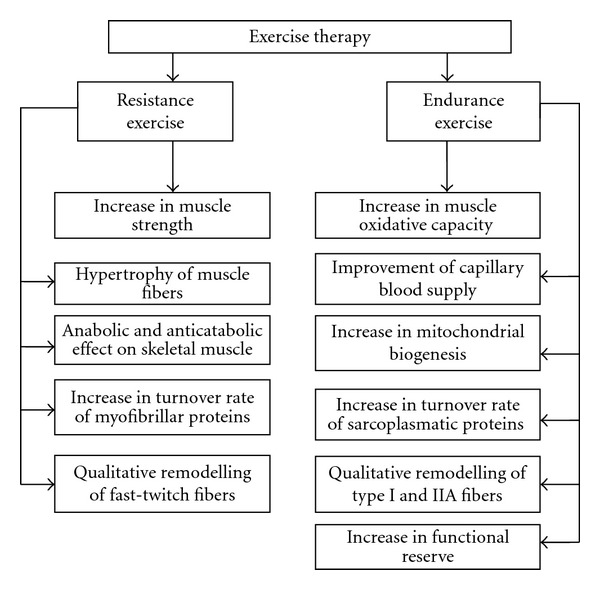
Effect of exercise therapy on aging, unloading, and glucocorticoid caused myopathic skeletal muscle.

Exercise therapy is a wide and systematic approach to the regular use of specific movements to improve different body functions, mobility, and fitness (Figure 2). Exercise therapy is a useful tool for the prevention and management of different injuries and diseases. On many occasions, specific exercise programs are tailored for rehabilitation needs. For example, in case of glucocorticoid caused myopathy, both endurance and strength exercise training has been shown to play a preventive role in the development of muscle atrophy, but a combination of both with different frequency, intensity and duration seems to be more effective (Figure 2).

More than four decades ago, the preventive role of exercise in the development of muscle atrophy during glucocorticoid administration was shown [25]. From the historical viewpoint, endurance exercise has been found to be an effective measure in retarding skeletal muscle atrophy associated with the administration of glucocorticoids [26, 29, 67]. From the contraction nature, four model systems have given the desired effect: endurance exercise, strength exercise, muscle functional overload, and in vitro cell culture stimulation [68]. Later intensive short-lasting exercise training has shown to have an anticatabolic effect on the contractile apparatus and the ECM of skeletal muscle [69]. Glucocorticoids increased myofibrillar protein degradation in FT muscles, while fibril- and network-forming collagen specific mRNA levels decreased at the same time in FT and ST muscles [45]. Both the myofibrillar apparatus and the ECM play a crucial role in changes of muscle strength during glucocorticoid administration and following muscle loading [70].

### 4.1. Effect of Resistance Exercise Training

Muscle atrophy contributes to but does not completely explain the decrease in force in the elderly. The age-related decrease in muscle mass and strength is a consequence of the complete loss of fibers associated with the decrease in the number of motor units and fiber atrophy [71]. In recent years, resistance exercise has become one of the fastest growing forms of physical activity for different purposes: improving athletic performance, enhancing general health and fitness, rehabilitation after surgery or an injury, or just for the pleasure of exercise [72]. Resistance exercise has shown to be an effective measure in the elderly, improving glucose intolerance, including improvements in insulin signaling defects, reduction in tumor necrosis factor-*α*, increases in adiponectin and IGF-1 concentrations, and reductions in total and abdominal visceral fat [73]. Resistance exercise improves skeletal muscle metabolism and through it muscle function in the elderly and their life quality [4].

Resistance exercise enhances the synthesis rate of myofibrillar proteins but not that of sarcoplasmic proteins [74] and this is related to mammalian target of rapamycin by activating proteins within the nitrogen-activated proteinkinase signaling [75]. A significant difference was observed between previously trained young and old participants in recovery from resistance training [76]. These results suggest a more rapid recovery in the young group. It seems that recovery from more damaging resistance exercise is slower as a result of age, whereas there are no age-related differences in recovery from less damaging metabolic fatigue [77].

It has been shown that resistance training, during which the power of exercise increased less than 5% per session, caused hypertrophy of both FT and ST muscle fibers, an increase of myonuclear number via fusion of satellite cells with damaged fibers or the formation of new muscle fibers as a result of myoblasts' fusion in order to maintain myonuclear domain size [78].

It has been shown that contractile proteins turned over faster in type I and IIA fibers than in IIB fibers and the turnover rate of skeletal muscle proteins in skeletal muscle depends on the functional activity of the muscle [30]. The turnover rate of myofibrillar proteins in aging skeletal muscle is related to the changes in MyHC isoforms' composition [4]. The effect of resistance training on the increase of the turnover rate of skeletal muscle contractile proteins in old age is relatively small [4]. Adaptational changes first appeared in newly formed or regenerating fibers and these changes lead to the remodeling of the contractile apparatus and an increase in the strength generating capability of muscle. These changes are more visible in muscle fibers with higher oxidative capacity. The recovery of locomotory activity after unloading is as fast as the recovery of muscle strength. It is related to the regeneration of muscle structure form disuse atrophy [79]. This fact suggests the presence of functionally immature muscle fibers during the recovery process following disuse atrophy [79]. So, the recovery of skeletal muscle mechanical properties depends on the structural and metabolic peculiarities of the skeletal muscle [30]. As a complex of factors contributes to the development of muscle wasting and weakness in the elderly, skeletal muscle unloading and glucocorticoid caused myopathy, it is complicated to find one certain measure for rehabilitation. As lack of strength is one of the central reasons for muscle weakness, it seems to be most realistic to use resistance training for this purpose in the elderly. Resistance training is a strong stimulus for muscle metabolism in the elderly, particularly for the contractile machinery of muscle (Figure 2).

### 4.2. Effect of Endurance Exercise Training

As oxidative capacity of skeletal muscle decreases in the elderly, endurance training is effective in stimulating mitochondrial biogenesis and improving their functional parameters [32, 33]. In combination with resistance training, the oxidative capacity and subsequently the turnover rate of contractile proteins in elderly skeletal muscle increases. This increase of the turnover rate of muscle proteins leads to the increase in skeletal muscle plasticity. It has recently been shown that the plasticity of individual development in the elderly makes it possible to modify the age-associated decline even in maximal physical performance [80]. Another positive influence of endurance training in the elderly is related to an increase in the ability of cardiovascular factors and to a lesser extent, to an increase in muscle mitochondrial concentration and capacity [81].

The increase in muscle oxidative capacity and contractile property is an effective measure for enhancing life quality in the elderly by improving skeletal muscle functional capacity and plasticity. It has recently been shown that the individual development of muscle plasticity in the elderly makes it possible to modify the age-associated decline even in maximal physical performance at least for some time [80]. The higher aerobic capacity in trained elderly people is related to an increase in the abilities of the cardiovascular system and to the lesser extent to an increase in muscle mitochondrial concentration [81]. It means that regular aerobic activity provides a foundation for an increase in muscle oxidative capacity in the elderly (Figure 2). It is useful to repeat the viewpoint of Suominen [80] that adequate physical performance is an essential element of a healthy and productive life among the elderly. Netz [82] studied the effect of physical activity on the moderating role of fitness improvement and mode of exercise to the potential mechanisms for explaining the physical activity affect relationship and found that neither improved fitness nor exercise modality serve as moderators of physical activity effect on affect. However, with older age managing everyday activities becomes less self-evident although there are gender differences in physical functioning [83]. Functional limitation is an objective measure of the consequences of disease and impairment [84].

It seems that the turnover rate of contractile proteins provides a mechanism by which the effect of exercise causes changes in accordance with the needs of the contractile apparatus. As the contractile protein turnover rate depends on the oxidative capacity of muscle and muscle oxidative capacity decreases in the elderly, it is obvious that endurance exercise stimulates an increase in the oxidative capacity of skeletal muscle by an increase in mitochondrial biogeneses and supports faster protein turnover during resistance training in order to increase muscle function (Figure 2). It has been shown that the aging-associated reduction in AMP-activated protein kinase (AMPK) activity may be a factor in reduced mitochondrial function [85]. In response to contractile activity, AMPK activation was registered only in aging FT muscles [86]. It is known that AMPK is activated in response to endurance exercise [87] and related to the metabolic adaptation of skeletal muscle. Later it has been shown that *α*1 isoform of AMPK is the regulator of skeletal muscle growth, but not of metabolic adaptation [66]. As factors such as health, physical function, and independence constitute components of quality of life in the elderly, physiological functioning of skeletal muscle in the elderly has significance in determining the ability to maintain independence and an active interaction with the environment [4, 88]. According to Kramer and Erickson [89], successful aging is guaranteed when elderly people use widespread participation in low-cost and low-tech exercise for further improving their fitness and reducing the risk of disability.

### 4.3. Effect of Concurrent Strength and Endurance Exercise Training

Concurrent training for strength and endurance has shown to decrease the gain in muscle mass in comparison with training for strength alone [90]. This effect was explained by AMPK blocking the activation of mammalian target of rapamycin complex-1 (TORC 1) by phosphorylating and activating the tuberous sclerosis complex-2 (TSC 2) [91]. This interference in skeletal muscle strength development was also explained by alterations in the protein synthesis induced by the high volume of endurance exercise or by frequent exercise training sessions [92] or was related to impairment of neural adaptations [93].

Concurrent strength and endurance exercise training in elderly men has shown that strength gain was similar to that observed with strength training alone, although strength training volume was half of that strength training alone [94]. Using lower training volumes in concurrent training in older men [95] in comparison with endurance and resistance training alone leads to similar strength enhancement with no presence of interference in this population [96]. In the elderly population, improvement in both strength and cardiorespiratory fitness is important and concurrent training is the best strategy to enhance cardiorespiratory fitness as it has widely been shown in the literature [93].

## 5. Conclusions and Future Directions

Changes in skeletal muscle mass and function with advancing age are reasons for disability in the aging population. Glucocorticoid treatment and skeletal muscle unloading lead to muscle atrophy, loss of myofibrillar proteins, changes in ECM and a decrease in muscle strength and motor activity. As a complex of factors supporting development of skeletal muscle wasting and weakness in the elderly in case of muscle unloading and glucocorticoid caused myopathy it is complicated to find one certain facility for rehabilitation. As the lack of strength is one of the central reasons in muscle weakness, it seems to be promising to use resistance training for this purpose in elderly. On the other hand, as oxidative capacity of skeletal muscle decreases in the elderly and endurance training is known to be an effective tool in the stimulation of mitochondrial biogenesis and improving their functional parameters, it seems that in combination with resistance exercise training, the oxidative capacity and subsequently the turnover rate of muscle contractile proteins in elderly skeletal muscle increases and leads to an increase in muscle plasticity. Physiological functioning of skeletal muscle in the elderly has significance in determining the ability to maintain independence and an active interaction with the environment.

Aging-associated reductions in AMPK activity may be a factor in the reduced mitochondrial function and AMPK is activated in response to endurance exercise, which explains the use of endurance exercise training in the prevention of disability and diseases. Frailty due to sarcopenia and dynapenia are proved reasons for the loss of an active interaction with the environment and loss of independence in the elderly, and predicate the use of resistance exercise for purposes of increasing muscle strength. The use of concurrent strength and endurance training in the prevention of muscle atrophy in the elderly population and the connection with anabolic and anticatabolic processes in skeletal muscle has not yet been elucidated. Future studies should focus on concurrent strength and endurance exercise effects on the prevention of skeletal muscle atrophy in the elderly during unloading and glucocorticoid treatment. The question is whether and in what conditions AMPK blocks the activation of TORC 1 by activating the TSC 2 during concurrent strength and endurance exercise in elderly skeletal muscle. To the best of our knowledge, it seems that concurrent training is an auspicious tool in the prevention or at least in the deceleration of the development of sarcopenia and dynapenia in the elderly. The important step is to optimize exercise therapy programs with respect to the interaction between signaling pathways for contractile and mitochondrial protein synthesis and degradation in the aging unloaded and myopathic population. This strategy to work out concurrent strength and endurance exercise programs in the elderly is complicated as it is unclear whether a muscle fiber is capable to undergo hypertrophy and maintain endurance capacity at the same time. It is clear that an exercise program has to be composed, which enables the recruitment of both ST and FT muscle fibers for the purpose of contributing the signaling pathways to accelerate protein turnover in aging skeletal muscle.
